# Clinical Characteristics and Treatment Patterns of Hyperhidrosis in Northern Hokkaido: A Single‐Center Retrospective Descriptive Study

**DOI:** 10.1111/1346-8138.70373

**Published:** 2026-07-01

**Authors:** Mari Kishibe, Aiko Noguchi, Marika Matsuya, Kou Matsumoto, Satomi Igawa, Yasuyuki Fujita

**Affiliations:** ^1^ Department of Dermatology Asahikawa Medical University Asahikawa Japan

**Keywords:** anticholinergic agent, hyperhidrosis, outpatient clinics, primary focal hyperhidrosis, sweating

## Abstract

Hyperhidrosis is a common condition associated with a substantial negative impact on the quality of life. Several studies have described the clinical characteristics and disease burden of patients with hyperhidrosis in Japan. However, comparable real‐world clinical data on hyperhidrosis from Hokkaido remain limited. We therefore evaluated real‐world clinical practice patterns at our institution before and after the establishment of a specialized outpatient clinic for hyperhidrosis. In this retrospective study, we analyzed the data of 144 patients who presented with hyperhidrosis between November 2013 and March 2025. Patient characteristics, pathways to care, treatment modalities, and treatment continuation were evaluated using data from electronic medical records. The sex distribution, age at presentation, and subtype distribution of hyperhidrosis in our cohort were largely consistent with those reported in a prior nationwide survey. Primary axillary and/or palmar hyperhidrosis accounted for 87.5% of all cases. After launching a specialized clinic in April 2023, we observed a substantial increase in the number of patients who consulted for hyperhidrosis during visits for other dermatologic conditions; this finding may suggest the presence of previously unrecognized patients. However, changes in hospital access policies may also have influenced consultation patterns. The treatment continuation rates for axillary and palmar hyperhidrosis were both approximately 40%. Among the treatments for axillary hyperhidrosis, sofpironium bromide gel was prescribed most frequently, whereas glycopyrronium tosylate hydrate wipes showed a higher treatment continuation rate. In conclusion, the presence of a specialized outpatient clinic may improve access to care for patients with primary focal hyperhidrosis and facilitate the identification of previously unrecognized patients. However, treatment continuation remained suboptimal, highlighting the importance of improving continuity of care in hyperhidrosis management at our institution.

## 1 Introduction

Sweating disorders, characterized by excessive or reduced sweating, are common conditions that substantially impair daily activities and quality of life. Among these disorders, hyperhidrosis is one of the most clinically important conditions because of its prevalence and the marked impact it has on the quality of life of patients [1, 2].

Several studies have described the clinical characteristics of patients with hyperhidrosis living in other regions of Japan, particularly Tokyo [3, 4]. However, comparable real‐world clinical data on hyperhidrosis from Hokkaido remain limited.

A dedicated outpatient clinic for sweating disorders was established at our hospital in April 2023 to provide specialized diagnostic and therapeutic care. At the time of its establishment, the number and clinical characteristics of patients with hyperhidrosis who attended this clinic were unknown, and the extent of regional demand for specialized hyperhidrosis care was unclear.

Therefore, in this study, we conducted a descriptive analysis of clinical data from patients with hyperhidrosis who visited a single‐center university hospital in Hokkaido to clarify their proportion, clinical characteristics, referral patterns, and management practices.

## 2 Methods

### 2.1 Study Population

This was a retrospective descriptive study. Patients who visited our university hospital with a chief complaint of hyperhidrosis between November 1, 2013, and March 31, 2025, were included. They were followed up until March 31, 2026. Cases were identified from the electronic medical record system using the diagnostic codes for primary focal hyperhidrosis (R610), generalized hyperhidrosis (R611), hyperhidrosis (R619), and bromhidrosis (L750) in accordance with the International Classification of Diseases, 10th Revision (ICD‐10) code. Patients suspected of having some form of hyperhidrosis (without a definitive diagnosis), those registered with diagnostic codes solely for insurance claims, and those with bromhidrosis alone were excluded.

### 2.2 Clinical Assessment of Patients With Hyperhidrosis

Clinical data were extracted from the medical records to characterize patients with hyperhidrosis, including diagnosis, sex, age, comorbidities, pathway to care (referral, opportunistic, or walk‐in visit), year of the first visit, duration of illness, affected sites, Hyperhidrosis Disease Severity Scale (HDSS) score, prior and initial treatment, and treatment outcomes. The year of the first visit was defined as the year in which the diagnostic code was first assigned.

Because treatment outcomes were not systematically documented in a substantial proportion of patients, treatment continuation was used as a pragmatic surrogate indicator of treatment engagement and effectiveness in real‐world clinical practice. Treatment continuation was defined as attending outpatient visits for ≥ 1 year, with either (i) at least two prescriptions for hyperhidrosis‐related medications within that period, (ii) receipt of other therapeutic interventions for hyperhidrosis, or (iii) documentation in the medical records confirming continued treatment. Patients in the discontinuation group were defined as those who did not meet these criteria; however, this classification reflected discontinuation of follow‐up at our hospital rather than confirmed treatment discontinuation.

Given that topical anticholinergic agents were introduced in 2020, the year of the first visit was included as a variable and categorized as before January 2020 or from January 2020 onward. Comorbidities were largely categorized into dermatological diseases, internal medicine conditions, neuropsychiatric disorders, and others. Treatment outcomes were assessed based on documentation in the medical records. Treatment efficacy was evaluated solely based on patient‐reported symptoms because post‐treatment HDSS data were unavailable in a substantial proportion of patients. For patients for whom documented evaluations were available, treatment was classified as either *effective* or *not effective*; for those without such documentation, treatment was classified as a missing value. Factors associated with treatment continuation were statistically evaluated by comparing the continuation and discontinuation groups.

Among patients with axillary and palmar hyperhidrosis, additional treatment‐level analyses were performed to evaluate the frequency of each treatment modality and treatment‐specific continuation.

### 2.3 Statistical Analysis

Statistical analyses were performed using Fisher's exact test or Mann–Whitney *U* test using EZR (Saitama Medical Center, Jichi Medical University), a graphical user interface for R [5].

Due to the retrospective nature of the study design, we considered that data for some variables might be missing for a subset of patients. Patients with missing values for the variables of interest were excluded from the analyses, and complete‐case analysis was performed. Statistical significance was set at *p* < 0.05.

## 3 Results

### 3.1 Study Population

**FIGURE 1:**
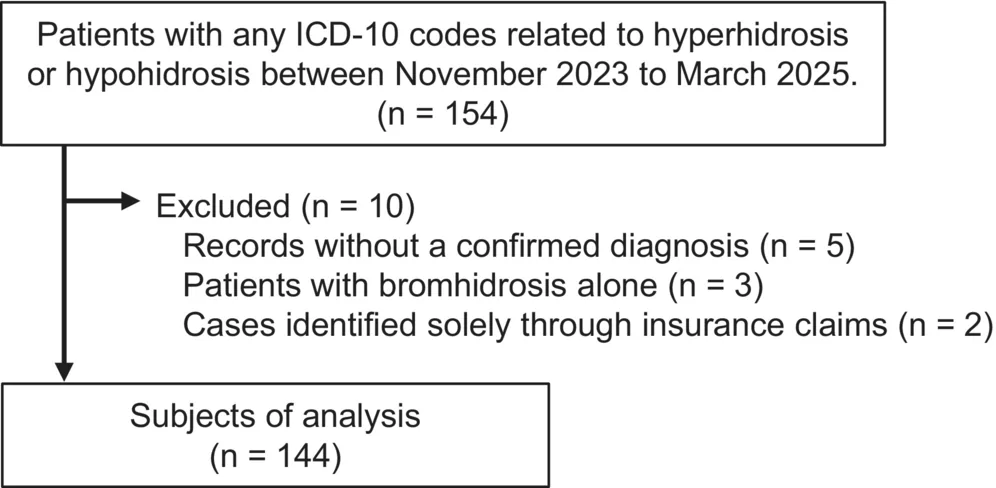
Flow chart of study population selection. ICD‐10, International Classification of Diseases, 10th revision.

During the study period, 154 patients visited our hospital with a chief complaint of hyperhidrosis (Figure 1). After excluding five patients without a confirmed diagnosis, three patients with bromhidrosis alone, and two patients identified solely through insurance claims, 144 patients with hyperhidrosis were included in the analysis.

### 3.2 Clinical Profile of Patients With Hyperhidrosis

**FIGURE 2:**
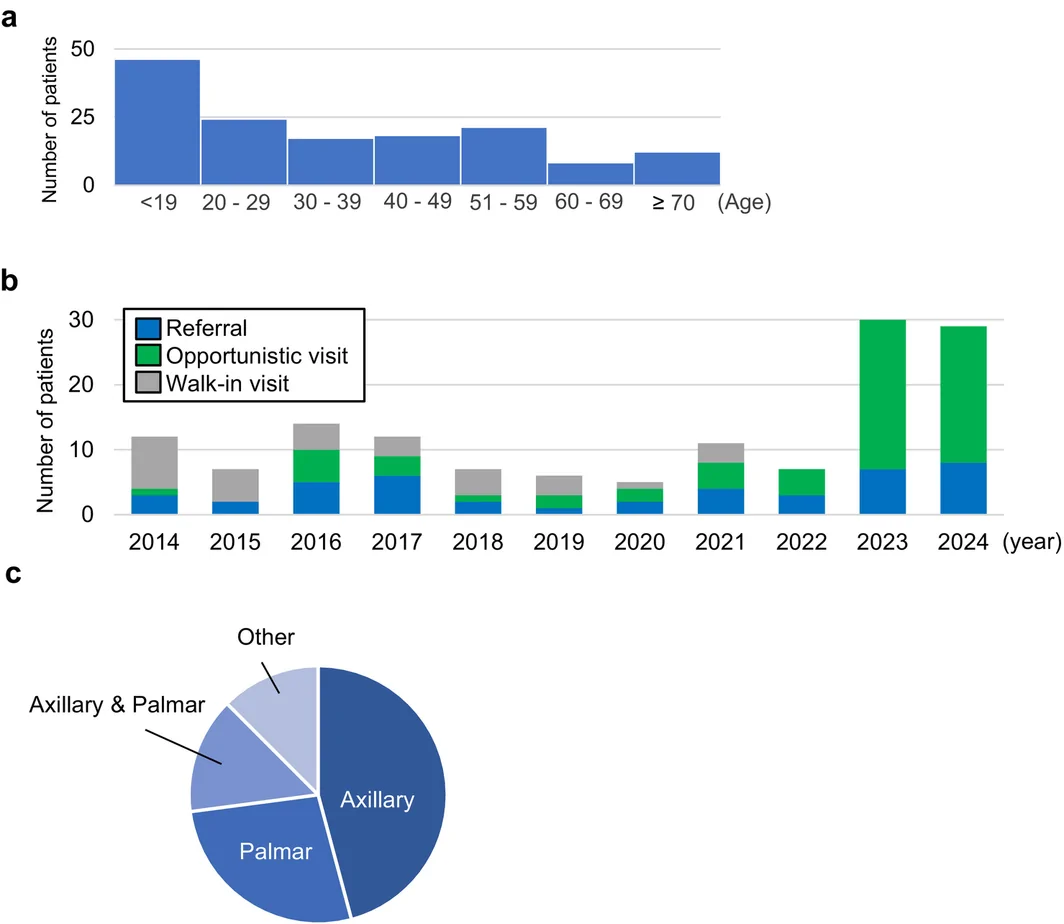
Clinical characteristics of patients with hyperhidrosis treated at our hospital. (a) Age at presentation, (b) annual number of patients and pathway to care, and (c) proportion of patients with primary axillary and palmar hyperhidrosis among all patients with hyperhidrosis.

Among patients diagnosed with hyperhidrosis, 48 were male and 96 were female, yielding a male‐to‐female ratio of approximately 1:2. The median age at presentation was 32.5 years, with most patients being in their teens (Figure 2a). The age at disease onset was available for 80 patients, with the median age of onset being 13.0 years.

### 3.3 Seasonal and Annual Trends in Patient Visits to Our Hospital for Hyperhidrosis

The distribution by the month of the first visit or initial consultation for hyperhidrosis. The number of visits began to increase around March, showed a temporary decrease in April, gradually increased toward August, and declined sharply in September.

The annual trends in the number of patients with hyperhidrosis are shown in Figure 2b. An additional cost for non‐referred outpatient visits was introduced in April 2022; thereafter, no walk‐in visits were observed. After the specialized outpatient clinic was opened in April 2023, the number of referred patients approximately doubled compared with the previous year. Opportunistic consultations for hyperhidrosis during visits for other dermatologic conditions increased approximately sevenfold.

Referral sources included dermatology clinics at other institutions (24 cases), pediatrics (6 cases), internal medicine (4 cases), general medicine (3 cases), and psychiatry, plastic surgery, and orthopedics (1 case each) departments. Overall, 27.8% of the patients were referred by non‐dermatologic departments.

### 3.4 Distribution of Affected Sites of Hyperhidrosis

Primary axillary and/or palmar hyperhidrosis accounted for 87.5% of all cases (Figure 2c). Briefly, the axilla was the most frequently affected site (87 cases, 60.4%), followed by the palm (60 cases, 41.7%). Among patients with axillary hyperhidrosis, 20 (23.0%) reported bromhidrosis. In patients with palmar hyperhidrosis, approximately 70% had concomitant plantar hyperhidrosis. Other affected sites included the craniofacial region (20 patients, 13.9%; overlapping cases included), entire body (8 patients, 5.6%), including one patient suspected of having secondary hyperhidrosis due to diabetes mellitus; and specific areas of the body (2 patients, 1.4%), including one patient with compensatory sweating following endoscopic thoracic sympathectomy.

### 3.5 Treatment Continuation and Associated Factors in the Overall Cohort

Given that the consultation rate for primary focal hyperhidrosis has been reported to be low [1, 2], we investigated whether treatment continuation at our hospital, a pragmatic surrogate indicator of treatment engagement and effectiveness, was also limited. Comparisons between the continuation and discontinuation groups are presented in Table 1. The treatment continuation rate was 37.5%.

**TABLE 1:** Descriptive characteristics of 144 patients with hyperhidrosis in the treatment continuation and discontinuation groups.

Descriptive characteristics	Continuation group	Discontinuation group	*p*
Number of the patients (% of all patients)	54 (37.5)	90 (62.5)	NA
Median age (IQR)	42.5 (23.3–55.8)	26.5 (16.3–48.3)	0.057
Sex			
Male, *n* (%)	12 (22.2)	36 (40.0)	0.030*
Female, *n* (%)	42 (77.8)	54 (60.0)	
Pathway to care			
Referral, *n* (%)	13 (24.1)	28 (31.1)	0.012*
Opportunistic visit, *n* (%)	34 (63.0)	35 (38.9)	
Walk‐in visit, *n* (%)	7 (12.9)	27 (30.0)	
Year of the first visit, *n* (%)			
Before December 2019	9 (16.7)	51 (56.7)	< 0.001*
January 2020 or later	45 (83.3)	39 (43.3)	
Duration of illness before first visit			
Duration of illness, years (IQR)	11.0 (6.3–29.0)	9.0 (5.0–10.0)	0.033
Missing values, *n* (%)	29 (53.7)	35 (38.9)	
Comorbid diseases (overlap included), *n* (%)			
Dermatological diseases	33 (61.1)	42 (46.7)	0.121*
Internal medicine conditions	16 (29.6)	7 (7.8)	< 0.001*
Neuropsychiatric disorders	4 (7.4)	10 (11.1)	0.570*
Others	2 (3.7)	1 (1.1)	0.556*
Distribution of affected sites (overlap included), *n*			
Axilla	35	52	0.406
Palmar	22	38	0.864
Plantar	15	27	0.779
Head and neck	11	9	0.083
Systemic	2	6	0.457
Localized	1	2	0.887
HDSS score at baseline, *n*			
4	11	6	0.341
3	34	27	
2	1	1	
1	0	1	
Missing values, *n* (%)	8 (14.8)	55 (61.1)	

Descriptive characteristics	Continuation group	Discontinuation group	*p*
Prior treatment (overlap included), *n* (%)			
No treatment	14 (25.9)	18 (20.0)	1**
Aluminum chloride use	3	10	
Over‐the‐counter medication	8	4	
Topical anticholinergic agents	2	1	
Oral propantheline	2	1	
Oral tofisopam	3	1	
Chinese herbal medicine	2	2	
Iontophoresis	0	2	
Botulinum toxin injection	0	2	
ETS	1	1	
Missing values	21 (38.9)	35 (38.9)	
Effectiveness of treatment			
Effective	42 (77.8)	12 (13.3)	< 0.001*
Not effective	0 (0)	40 (44.4)	
Missing value	12 (22.2)	35 (42.2)	
Follow‐up periods at our hospital			
Duration follow‐up periods, month (IQR)	31 (22–60)	4 (2–18)	< 0.001

*Note: p* Value were calculated using the nonparametric Mann–Whitney *U* test. Cases with missing values for the variables of interest were excluded from the analyses, and complete‐case analysis was performed.

Abbreviations: ETS, endoscopic thoracic sympathectomy; HDSS, Hyperhidrosis Disease Severity Scale; IQR, interquartile range; *n*, number of patients; NA, not applicable.

* *p* values were calculated using Fisher's exact test.

** Comparisons between patients with any treatment and those without treatment were performed using Fisher's exact test.

We performed univariable statistical analyses (Fisher's exact and Mann–Whitney *U* tests) to identify factors associated with treatment continuation. Sex differed significantly between the two groups, whereas age did not. At our hospital, the pathway to care was significantly associated with treatment continuation. The proportion of patients with opportunistic visits was higher in the continuation group, whereas the proportion of referral and walk‐in visits was higher in the discontinuation group. Furthermore, the proportion of patients who first attended the clinic in 2020 or later was significantly higher in the continuation group. The median duration of illness before the first visit was 11.0 years in the continuation group and 9.0 years in the discontinuation group, with the between‐group difference being significant; however, data for a substantial proportion of the cohort were missing.

A significant between‐group difference was observed only for internal medicine comorbidities. When specific conditions were examined (Table S1), connective tissue diseases were more prevalent in the continuation group (Fisher's exact test, *p* < 0.001), whereas infectious diseases of the skin were more frequently observed in the discontinuation group (Fisher's exact test, *p* = 0.011).

No significant difference in affected sites, HDSS score, or prior treatments was noted between the two groups. The HDSS score and prior treatment data are summarized in Table 1; however, a substantial amount of data was missing, the sample sizes were small in several categories, and treatment exposures overlapped. These may have caused violations of the assumptions of standard statistical tests.

Treatment efficacy was more frequently documented in the continuation group; both patients without documented treatment efficacy and those without documented assessment of treatment efficacy (treated as missing values) were similarly common in the discontinuation group.

At our hospital, the follow‐up period was significantly longer in the continuation group than in the discontinuation group.

We conducted a similar analysis involving 126 patients with axillary and palmar hyperhidrosis. Significant differences in sex, pathway to care, year of the first visit, treatment efficacy, and follow‐up period were also observed between the continuation and discontinuation groups in this subgroup (Table 2).

**TABLE 2:** Descriptive characteristics of 126 patients with axillary and palmary hyperhidrosis in the treatment continuation and discontinuation groups.

Descriptive characteristics	Continuation group	Discontinuation group	*p*
Number of the patients (% of all patients)	47 (37.3)	79 (62.7)	NA
Median age (IQR)	39.0 (19.5–52.5)	24.0 (16.0–45.5)	0.082
Sex			
Male, *n* (%)	9 (19.1)	31 (39.2)	0.029
Female, *n* (%)	38 (80.9)	48 (60.8)	
Pathway to care			
Referral, *n* (%)	9 (19.1)	22 (27.8)	0.008
Opportunistic visit, *n* (%)	32 (68.1)	32 (40.5)	
Walk‐in visit, *n* (%)	6 (12.8)	25 (31.6)	
Year of the first visit, *n* (%)			
Before December 2019	9 (19.1)	45 (57.0)	< 0.001
January 2020 or later	38 (80.9)	34 (43.0)	
Effectiveness of treatment			
Effective	37 (78.7)	37 (46.8)	0.002
Not effective	0 (0)	10 (12.6)	
Missing value	10 (21.3)	32 (40.5)	
Follow‐up periods at our hospital			
Duration of follow‐up periods, month (IQR)	37 (22–61)	5 (2–20)	< 0.001

*Note: p* value were calculated using Fisher's exact test. Cases with missing values for the variables of interest were excluded from the analyses, and complete‐case analysis was performed.

Abbreviations: IQR, interquartile range; *n*, number of patients; NA, not applicable.

### 3.6 Reasons for Treatment Discontinuation at Our Hospital

The reasons for treatment discontinuation at our hospital were summarized based on data in the medical records (Table 3). More than half the patients were lost to follow‐up, and the reasons for their discontinuation of treatment were not documented. Among the documented reasons, difficulty attending appointments due to work or school and transfer to another clinic were the most common. Other reasons included undergoing endoscopic thoracic sympathectomy, distance from the hospital, comorbid conditions, high treatment costs, improvement in symptoms, dissatisfaction with treatment outcomes, initiation of home iontophoresis, and adverse events related to topical anticholinergic agents.

**TABLE 3:** Reasons for treatment discontinuation among 90 patients with hyperhidrosis in the discontinuation groups.

Reason	Number (%)
Lost to follow‐up (reason not documented)	50 (55.6)
Difficulty attending appointments due to work or school	10 (11.1)
Transfer to another clinic	9 (10.0)
Underwent endoscopic thoracic sympathectomy	4 (4.4)
Difficulty attending appointments due to distance from the hospital	4 (4.4)
Difficulty attending appointments due to comorbid conditions	3 (3.3)
High treatment costs	3 (3.3)
Improvement in symptoms	2 (2.2)
Dissatisfaction with the treatment results	2 (2.2)
Initiation of home iontophoresis	2 (2.2)
Adverse events related to topical anticholinergic agents	1 (1.1)

*Note:* Percentages were calculated based on the total number of patients in the discontinuation group.

### 3.7 Treatment Modalities and Continuation Rates in Patients With Axillary and Palmar Hyperhidrosis

**FIGURE 3:**
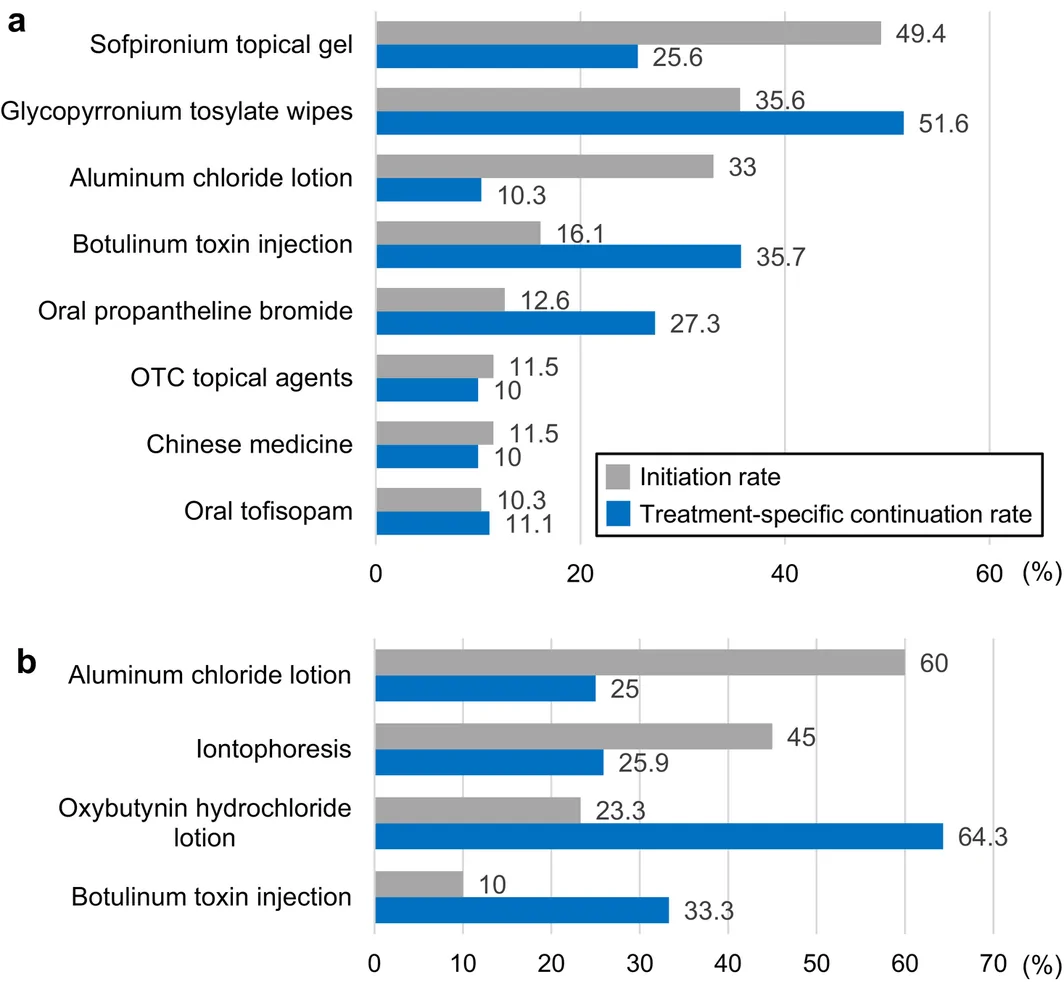
Treatment initiation rates and treatment‐specific continuation rates by modality for (a) axillary hyperhidrosis and (b) palmar hyperhidrosis. Overlapping treatments were allowed. Gray and blue bars indicate treatment initiation and continuation rates, respectively. OTC, over‐the‐counter.

Separate from the patient‐level analysis described above, we conducted a descriptive analysis of treatment‐level outcomes, including the frequency of each treatment modality and treatment‐specific continuation rates in patients with axillary and palmar hyperhidrosis. No formal statistical comparisons were performed because of overlapping treatment exposures. Among the treatments for axillary hyperhidrosis, sofpironium bromide gel was prescribed most frequently, followed by glycopyrronium tosylate hydrate wipes (Figure 3a). However, the highest treatment continuation rate was observed with glycopyrronium tosylate hydrate wipes (51.6%), followed by botulinum toxin type A injections (35.7%) and sofpironium bromide gel (25.6%). Although 20% aluminum chloride lotion ranked third in prescription frequency, it demonstrated a notably low continuation rate (10.3%).

Treatment‐specific continuation rates and reasons for discontinuation of topical anticholinergic therapies in patients with axillary hyperhidrosis are shown in Table 4. Both treatments were frequently discontinued due to loss to follow‐up. Among patients, 23.3% initially treated with sofpironium bromide gel switched to glycopyrronium tosylate wipes, primarily due to treatment preference. Conversely, patients initially treated with glycopyrronium tosylate wipes were more likely to switch to therapies other than topical anticholinergics, such as botulinum toxin injection or endoscopic thoracic sympathectomy.

**TABLE 4:** Treatment initiation, treatment‐specific continuation, and reasons for discontinuation of topical anticholinergic therapies in 87 patients with axillary hyperhidrosis (overlapping treatments allowed).

Treatment	Treatment initiation, *n* (% of all patients)	Treatment outcomes, *n* (% of treated patients)
Sofpironium bromide gel	43 (49.4)	Continued treatment, 11 (25.6) Switched to glycopyrronium tosylate wipes, 10 (23.3) Treatment preference, 8 Adverse events, 1 Insufficient treatment effectiveness, 1 Discontinued due to difficulty attending appointments, 2 (4.7) Discontinued due to adverse events, 1 (2.3) Discontinued due to improvement in symptoms, 1 (2.3) Switched to therapies other than topical anticholinergics, 1 (2.3) Lost to follow‐up, 17 (39.5)
Glycopyrronium tosylate wipes	31 (35.6)	Continuation, 16 (51.6) Switched to therapies other than topical anticholinergics, 4 (12.9) Switched to sofpironium bromide gel, 2 (6.4) Treatment preference, 1 Insufficient treatment effectiveness, 1 Discontinued due to adverse events, 1 (3.2) Transfer to another hospital, 1 (3.2) Lost to follow‐up, 7 (22.6)

Abbreviation: *n*, number of patients.

For palmar hyperhidrosis, while topical 20% oxybutynin hydrochloride lotion was the third most frequently prescribed medication, it showed the highest continuation rate (64.3%; Figure 3b). Botulinum toxin type A injections were only administered to 10.0% of patients because the treatment was not covered by national health insurance; however, the continuation rate (33.3%) was comparable to that of iontophoresis.

## 4 Discussion

In this study, we systematically analyzed the real‐world clinical practice and patterns for seeking healthcare at our hospital, revealing previously uncharacterized unmet needs in the management of hyperhidrosis. Our findings demonstrated that the establishment of a specialized outpatient clinic has improved access to care for patients with primary focal hyperhidrosis. However, low treatment continuation rates at our hospital remain a key challenge. Because this study was designed as a descriptive analysis to characterize the clinical features observed at our hospital setting rather than to establish causal relationships, the findings should be interpreted accordingly.

The absolute number of patients, particularly those with hyperhidrosis, in our study was substantially lower than that reported in studies involving university hospitals in Tokyo and regions south of Tokyo. For example, at Tokyo Medical and Dental University (now the Institute of Science Tokyo), 284 patients with hyperhidrosis alone were evaluated over a 3‐year period. Forty‐three patients with sweating disorders, including 26 patients with hyperhidrosis (60.5%), visited a specialized outpatient clinic at Nagasaki University within its first year of operation [3, 4].

Notably, despite the smaller number of patients, the sex distribution, age at presentation, and subtype distribution of hyperhidrosis in our cohort were largely consistent with those reported in a prior nationwide survey [6]. These findings suggest that, rather than inherently different in their clinical characteristics, hyperhidrosis in the clinical region served by our hospital is more likely to be under‐recognized and ‐referred. The relatively small number of patients likely reflects a combination of regional population size, disease awareness, and accessibility to specialized care, in contrast to metropolitan areas such as Tokyo.

A seasonal variation was observed, with visits for hyperhidrosis increasing from spring through summer and declining sharply after September. Associations between the number of visits for hyperhidrosis and meteorological parameters, including ambient and apparent temperature, have been demonstrated [7]. Although indoor environments in Hokkaido are typically maintained at relatively high temperatures during winter [8, 9], the impact of indoor environmental factors on sweating and healthcare‐seeking behavior remains unclear. Further investigation is warranted.

Despite the substantial economic, psychological, and social burdens associated with primary focal hyperhidrosis, disease awareness and healthcare utilization remain low [1, 2]. Previous surveys in Japan have demonstrated a marked discrepancy between disease prevalence and consultation rates, with only a small proportion of affected individuals seeking medical care [6, 10]. Furthermore, a previous report has shown that limited access to specialized care, reliance on over‐the‐counter products, and dissatisfaction with the treatments that are available may impede healthcare utilization among patients with hyperhidrosis [11]. After our specialized outpatient clinic was launched, we observed a substantial increase in the number of patients who consulted incidentally for hyperhidrosis during visits for other dermatologic conditions; this may suggest the presence of previously unrecognized patients. These findings are consistent with the concept of *latent hyperhidrosis* and suggest that structured clinical pathways may lower barriers to care. Notably, approximately 30% of patients were referred to our department from non‐dermatologic specialty departments, indicating that some patients may have difficulty identifying appropriate points of care. Establishing a specialized outpatient clinic may have contributed to improved awareness of hyperhidrosis among patients as well as healthcare providers, thereby facilitating appropriate referral and earlier intervention. However, changes in hospital access policies introduced in April 2022, including additional fees for non‐referred outpatient visits, may also have contributed to the observed changes in consultation patterns. Therefore, the relative contributions of these factors cannot be determined from the present study.

In our cohort, axillary and palmar hyperhidrosis accounted for most cases, highlighting a high demand for treatment of these subtypes. Consistent with a previous report [11], symptom onset typically occurred during adolescence, and a substantial proportion of patients were referred from pediatric departments. These findings highlight the importance of strengthening collaboration with pediatricians and establishing transitional care pathways.

A previous study showed that the most common comorbidities associated with hyperhidrosis include diabetes mellitus, thyroid disease, nonspecific joint and bone pain, cardiovascular disease, and neuropsychiatric disorders [12]. Patients in the continuation group in our cohort were more likely to have concomitant internal medicine conditions, particularly connective tissue diseases. This association may not indicate a direct link between hyperhidrosis and these conditions but rather reflect the fact that patients with connective tissue diseases requiring regular follow‐up at a university hospital are more likely to continue treatment for hyperhidrosis. This may partly contribute to the longer follow‐up period observed in the continuation group. These findings suggest that, in our hospital setting, maintaining treatment solely for hyperhidrosis may be challenging in routine clinical practice.

The treatment continuation rate for hyperhidrosis, which was largely driven by axillary and palmar hyperhidrosis, was relatively low and associated with the year of the first visit, treatment efficacy, and follow‐up period. Since 2020, several topical anticholinergic agents have been approved in Japan, and their efficacy and safety have been demonstrated in phase III clinical trials [13, 14, 15, 16, 17]. In our practice, although sofpironium bromide gel was the most frequently prescribed agent, better treatment continuation was achieved with glycopyrronium tosylate hydrate wipes. Although no study has directly compared the efficacy and safety of sofpironium bromide gel and glycopyrronium tosylate hydrate wipes, clinical trials showed that the two agents have broadly comparable efficacy and similar frequencies of treatment‐related adverse events [13, 14, 15]. One possible explanation for the higher continuation rate observed with glycopyrronium tosylate hydrate wipes is differences in formulation characteristics, which may influence usability and convenience relative to sofpironium bromide gel. In our study, the primary reason for switching from sofpironium bromide gel to glycopyrronium tosylate wipes was patient preference. Medical records indicated that some patients described the wipes as convenient to carry and expressed a preference for continuing treatment because of the favorable sensory characteristics during and after application of the wipes. However, because this was a retrospective study based on a review of medical records, we were unable to evaluate patient‐related factors such as usability, treatment satisfaction, perceived efficacy, or adverse events in sufficient detail. Furthermore, because treatment continuation was defined according to follow‐up status, some patients classified into the discontinuation group may have experienced symptom improvement, transferred their care elsewhere, undergone alternative interventions, or discontinued follow‐up for reasons unrelated to treatment failure. Given the small sample size and descriptive nature of this analysis, classification into the discontinuation group should be interpreted with caution and should not be considered confirmation of treatment discontinuation. Further prospective studies incorporating patient‐reported assessments are warranted.

A similar trend was observed in palmar hyperhidrosis; Oxybutynin hydrochloride lotion, a topical anticholinergic agent that is suitable for patients aged 12 years and older, was associated with the highest continuation rate, despite aluminum chloride preparations frequently being used as the initial treatment in younger patients.

Treatment outcomes were not systematically documented in a substantial proportion of patients. This likely reflects variability in routine clinical practice and, in part, the limited opportunity to assess patients who discontinued follow‐up. Given the descriptive nature of this study, residual confounding cannot be excluded. Our findings nevertheless highlight the importance of proactive assessment and systematic documentation of treatment responses in routine clinical practice. Developing and implementing standardized assessment tools, including patient‐reported outcome measures, may improve both clinical evaluation and patient satisfaction [17, 18]. Enhanced patient–physician communication, multidisciplinary collaboration, and incorporation of patient preferences into treatment decisions may further reduce treatment discontinuation. Importantly, these principles should be integrated into physician education to ensure sustainable improvements in the management of hyperhidrosis.

This study had some limitations. First, as a single‐center study conducted at a university hospital, referral bias toward more severe or diagnostically complex cases cannot be excluded, which may limit the generalizability of the findings. Second, the retrospective design relied on a review of medical records, resulting in the incompleteness of some patients' data, including substantial missing data for the HDSS score, prior treatment before presentation to our hospital, and assessments of treatment effectiveness. Third, because this study included only patients who visited during the study period, the cohort may be inherently biased toward individuals who were able to continue treatment, representing a potential selection bias. Fourth, treatment continuation was defined according to follow‐up status at our hospital rather than confirmed treatment status. Therefore, some patients classified into the discontinuation group may not have actually discontinued treatment, which may have affected the interpretation of treatment continuation analyses. Finally, this study was primarily descriptive in nature, aiming to characterize clinical characteristics and management patterns at a single center. Therefore, multivariable analyses were not performed, and causal relationships could not be established.

In this study, consultations for hyperhidrosis increased after the establishment of a specialized outpatient clinic, including consultations by patients who previously may not have been recognized. These findings suggest a potential role for structured care pathways in facilitating access to care and disease recognition within our hospital setting. However, treatment continuation rates for the predominant subtypes of axillary and palmar hyperhidrosis remained suboptimal. Topical anticholinergic agents may offer a promising option to improve treatment adherence.

Further investigations regarding the roles of standardized outcome assessment and proactive documentation of treatment response are needed; however, our findings highlight the importance of improving continuity of care in hyperhidrosis management. Moreover, strengthening collaboration across clinical departments, establishing referral networks, and fostering physician expertise in hyperhidrosis may further support sustainable care. Collectively, these efforts may help address unmet needs in the long‐term management of hyperhidrosis at our hospital.

## Funding

This work was supported by The Ministry of Health, Labour and Welfare (MHLW) Research Program on Rare and Intractable Diseases (25FC1014).

## Ethics Statement

Approval of the research protocol by an institutional review board: This study was reviewed and approved by the Ethics Committee of Asahikawa Medical University (approval number 25067).

## Consent

The authors have nothing to report.

## Conflicts of Interest

M.K. is an editorial board member of The Journal of Dermatology. To minimize bias, M.K. was excluded from all editorial decision‐making regarding the publication of this article. Y.F. has received research grants from Maruho Co. Ltd. and Kaken Pharmaceutical Co. Ltd., and has received speaker fees from Maruho Co. Ltd.

## Supporting information


**Table S1:** Comorbid diseases of patients with hyperhidrosis in the treatment continuation and discontinuation groups.

## Data Availability

The data that support the findings of this study are available on request from the corresponding author. The data are not publicly available due to privacy or ethical restrictions.
