# Supplementary material for: Clinical Characteristics and Treatment Patterns of Hyperhidrosis in Northern Hokkaido: A Single‐Center Retrospective Descriptive Study
Source: J Dermatol. 2026 Jul 1;53(9):1267–76. doi: 10.1111/1346-8138.70373 (PMC13555164; doi:10.1111/1346-8138.70373)
Supplement: Supplementary file 1 [file JDE-53-1267-s001.docx]

**Clinical Characteristics and Treatment Patterns of Hyperhidrosis in Northern Hokkaido: A Single-Center Retrospective Descriptive Study**

Mari Kishibe, Aiko Noguchi, Marika Matsuya, Kou Matsumoto, Satomi Igawa, Yasuyuki Fujita

**Supporting Information**

Supplementary Table 1. Comorbid diseases of patients with hyperhidrosis in the treatment continuation and discontinuation groups.

**Supplementary Table 1. Comorbid diseases of patients with hyperhidrosis in the treatment continuation and discontinuation groups.**

| **Comorbid disease** | **Continuation group** | **Discontinuation group** |
| --- | --- | --- |
| Dermatological diseases | 16 Eczema and dermatitis  7 Seborrheic dermatitis  2 Atopic dermatitis  2 Hand eczema  2 Contact dermatitis  2 Dyshidrotic dermatitis  2 Others  5 Acne  4 Nevus  4 Palmoplantar keratoderma  2 *SERPINB7*-pEED  2 Unclassified  4 Urticaria  3 Alopecia  3 Palmoplantar pustulosis  3 Rosacea  3 Senile lentigines  3 Infections  1 Folliculitis  1 Tinea  1 Warts  3 Melasma  1 Calluses  1 Chilblains  1 Basal cell carcinoma  1 Female-pattern hair loss  1 Hand–foot syndrome  1 *FLG-*nEDD  1 Ingrown nail  1 Telangiectasia | 11 Infections  5 Tinea  2 Herpes zoster  2 Folliculitis  1 Herpes simplex  1 Warts  12 Eczema and dermatitis  6 Atopic dermatitis  2 Seborrheic dermatitis  2 Contact dermatitis  1 Dyshidrotic dermatitis  1 Others  8 Urticaria  8 Acne  6 Senile lentigines  3 Rosacea  2 Alopecia  2 Basal cell carcinomas  2 Melasma  2 Psoriasis  2 *SERPINB7*-pEDD  1 Calluses  1 Chilblains  1 Epidermoid cyst  1 Food-dependent exercise induced anaphylaxis  1 Keratosis pilaris  1 Keloids  1 Postinflammatory hyperpigmentation  1 Pruritus  1 Telangiectasia  1 Vitiligo |
| Internal Medicine Conditions | 6 SLE  2 Behçet’s disease  2 Hypothyroidism  1 Asthma  1 Crohn’s disease  1 Diabetes  1 Eosinophilic granulomatosis with polyangiitis  1 Mixed connective tissue disease  1 Primary biliary cholangitis  1 Sjögren’s syndrome  1 Systemic sclerosis  1 Zinc deficiency | 3 Diabetes  2 Hypertension  1 Congestive heart failure  1 Dyslipidemia  1 Hyperthyroidism  1 Idiopathic thrombocytopenic purpura  1 Immunoglobin A nephropathy  1 Renal failure |
| Neuropsychiatric disorders | 2 Developmental disorders  1 Anxiety disorders  1 Autonomic dysfunction  1 Epilepsy  1 Learning disability | 3 Autism spectrum disorder  3 Attention-deficit/hyperactivity disorders  2 Autonomic dysfunctions  2 Somatoform disorder  1 Adjustment disorder  1 Alcoholic amnesia  1 Congenital ataxia  1 Insomnia  1 Menopausal disorder  1 Mental retardation  1 Obsessive–compulsive disorder  1 Old cerebral infarction  1 Panic disorder |
| Others | 1 Benign prostatic hyperplasia  1 Complex regional pain syndrome | 1 Spinal stenosis |

*SERPINB7*, serine protease inhibitor B7; pEDD, palmoplantar epidermal differentiation disorders; *FLG*, filaggrin; nEDD, non-syndromic epidermal differentiation disorder; SLE, systemic lupus erythematosus.
